# A novel interactive mobile health support system for pediatric obesity treatment: a randomized controlled feasibility trial

**DOI:** 10.1186/s12887-020-02338-9

**Published:** 2020-09-23

**Authors:** Linnea Johansson, Emilia Hagman, Pernilla Danielsson

**Affiliations:** 1 Department of Clinical Science, Intervention and Technology, Division of Pediatrics, Karolinska Institutet, CLINTEC, Novum, Blickagangen 6A, 141 57 Huddinge, Sweden; 2 Health Professionals Function, Medical Unit Occupational Therapy & Physiotherapy, Karolinska University Hospital, Stockholm, Sweden

**Keywords:** Mobile health, Self-monitoring, Pediatric obesity, Feasibility, Obesity treatment

## Abstract

**Background:**

In order to achieve improved weight status, behavioral pediatric obesity treatment is resource intensive. Mobile Health (mHealth) is more accessible than standard care but effective approaches are scarce. Therefore, the aim of this feasibility trial was to study trial design, mHealth usage, compliance, and acceptability of a novel mHealth approach in pediatric obesity treatment.

**Methods:**

This six-month parallel two-arm feasibility trial took place at three pediatric outpatient clinics in Stockholm, Sweden. Participants, 5–12 years, starting obesity treatment were randomized to using an mHealth support system as an addition to standard care (intervention) or to standard care alone (control). The intervention included daily self-monitoring of weight transferred to a mobile application (app) used by parents, a website in which clinicians could track treatment progress, prespecified treatment goals for change in degree of obesity shown in the app and on the website, and text message interactions between clinicians and parents. The main outcome was description of feasibility. Height and weight were measured at baseline, three, and 6 months to explore changes in body mass index standard deviation score (BMI SDS).

**Results:**

Of 40 children eligible for inclusion, 28 agreed to participate (54% girls) and were randomized to intervention (*n* = 15) or control (*n* = 13). Weight was measured at home regularly throughout the entire trial period by 12/15 children in the intervention group. Attendance at appointments were better in the intervention group (*p* = 0.024). Both parents and clinicians had a positive experience and found the mHealth support system accessible. At 6 months the intervention group had a greater reduction of 0.24 units in BMI SDS than standard care (− 0.23 vs. 0.01, *p* = 0.002).

**Conclusions:**

The mHealth support system was a feasible and innovative treatment approach which, in addition to standard care, generated better treatment results than standard care alone. Future research should evaluate the treatment effects over a longer follow-up time in a larger study sample.

**Trial registration:**

This trial was retrospectively registered at ClinicalTrials.gov, ID: NCT03380598, on November 8, 2017.

## Background

The prevalence of childhood obesity in Sweden has been estimated to 4% to 9% during the last decade [1] and the number of children receiving obesity treatment increases annually [2]. The treatment of pediatric obesity is resource intensive and there is an association between the intensity, parental involvement, and the outcome of treatment [3–5]. Another challenge is the drop-out rate in clinical obesity treatment programs, which has been shown to range from 27 to 73% [6]. Frequent appointments, including extensive travelling time, entail high levels of absence from school and work for the families involved, and easy access to pediatric clinics appears to be important to reduce attrition [7, 8].

Due to the increased availability of smartphones and their convenience, mobile Health (mHealth) has become more common in several healthcare disciplines [9]. Mobile Health provide flexibility for the user and includes mobile applications (apps), text messages or wearable monitoring devices. In pediatric obesity treatment, several mHealth approaches have been positively received by children and their parents [10, 11]. Nevertheless, the evaluated mHealth approaches appear to be less effective regarding changes in weight, diet and physical activity (PA) and the interventions commonly focus on self-reported rather than objectively measured data [10, 12–14]. In both children and adults with obesity, self-monitoring have shown positive effects on weight outcomes [15–17] and in a meta-analysis by Darling & Sato, 14 studies were included in which mHealth interventions had a self-monitoring aspect. The results showed small effect sizes of weight (*d* = 0.41), diet (*d* = 0.10) and PA (*d* = 0.42) and none of the interventions tracked self-monitored objective data [12]. Since self-reported data regarding diet, physical activity, and weight lack of validity [18–20], objective data are important for increased accuracy.

When measuring weight in children, there are several important aspects to consider. As children grow taller, they can gain weight (kg) and still reduce their degree of obesity (BMI SDS). Therefore, an outcome measure reflecting the relative weight change, e.g. body mass index standard deviation score (BMI SDS), should be used rather than the weight in kilograms. For many individuals BMI SDS is difficult to understand, and it is therefore vital to facilitate the understanding of the treatment outcome. To the best of our knowledge, no mHealth intervention for pediatric obesity treatment has previously combined the following components: a) daily self-monitoring of weight, b) a mobile app used by parents, displaying objectively measured change in degree of obesity in relation to a prespecified treatment goal, c) a website on which clinicians view the same data as parents do in the mobile app and d) communication between clinicians and parents by text messages through the website and the mobile app.

This randomized feasibility trial included an intervention group using an mHealth support system as an addition to standard care and a control group receiving standard care alone. The aim was to study feasibility in terms of trial design, mHealth usage, compliance, and acceptability of the treatment from parents and clinicians. The primary objectives were:

1. To study trial design in terms of recruitment process and attrition rates
2. To study mHealth usage regarding frequency of measured weights and sent messages
3. To study compliance with treatment in both the intervention and control group in terms of attendance at appointments
4. To study parental acceptability in terms of parental experience of treatment in both the intervention and the control group

Secondary objectives were:

1. To study clinicians’ acceptability of treatment in terms of required working time and user experience of the mHealth intervention
2. To explore the response in BMI SDS in the intervention and the control group after three and 6 months

## Methods

### Trial design

This was a parallel open-label randomized controlled feasibility trial in which two treatment approaches for children with obesity were studied over the course of 6 months. The study was approved by the Regional Ethical Committee in Stockholm, Sweden no. 2017/667–31/5 and registered at ClinicalTrials.gov, ID: NCT03380598. The trial adheres to the CONSORT guidelines 2010: extension to randomized pilot and feasibility trials.

### Participants

Participants were referred, from either the Primary child health care or the School health care, for obesity treatment to any of three outpatient pediatric clinics in Stockholm county between September 2017 and February 2018. Children were recruited consecutively by the clinicians at their first appointment to the pediatric clinic. If a family was interested, the researchers informed the parents about the study and obtained their written informed consent together with written assent from their child.

The inclusion criteria were: a) age 5 to 12 years, b) obesity according to the International Obesity Task Force (IOTF) [21], c) parents speaking Swedish, d) parents being able to use a smartphone, and e) no pharmacological treatment affecting the obesity intervention. The exclusion criteria were: a) diagnosed or ongoing assessment of neuropsychiatric disorder, b) obesity treatment during the last 6 months, and c) hypothalamic obesity.

Children were randomly allocated to the support system in combination with standard care (intervention) or to standard care alone (control) using sealed coded envelopes selected by a researcher not involved in this study. Three block randomizations (1:1) were performed, one at each pediatric outpatient clinic. This study was explorative. Thus, no power estimation was performed.

### Treatment models and settings

#### The mHealth support system

**Fig. 1:**
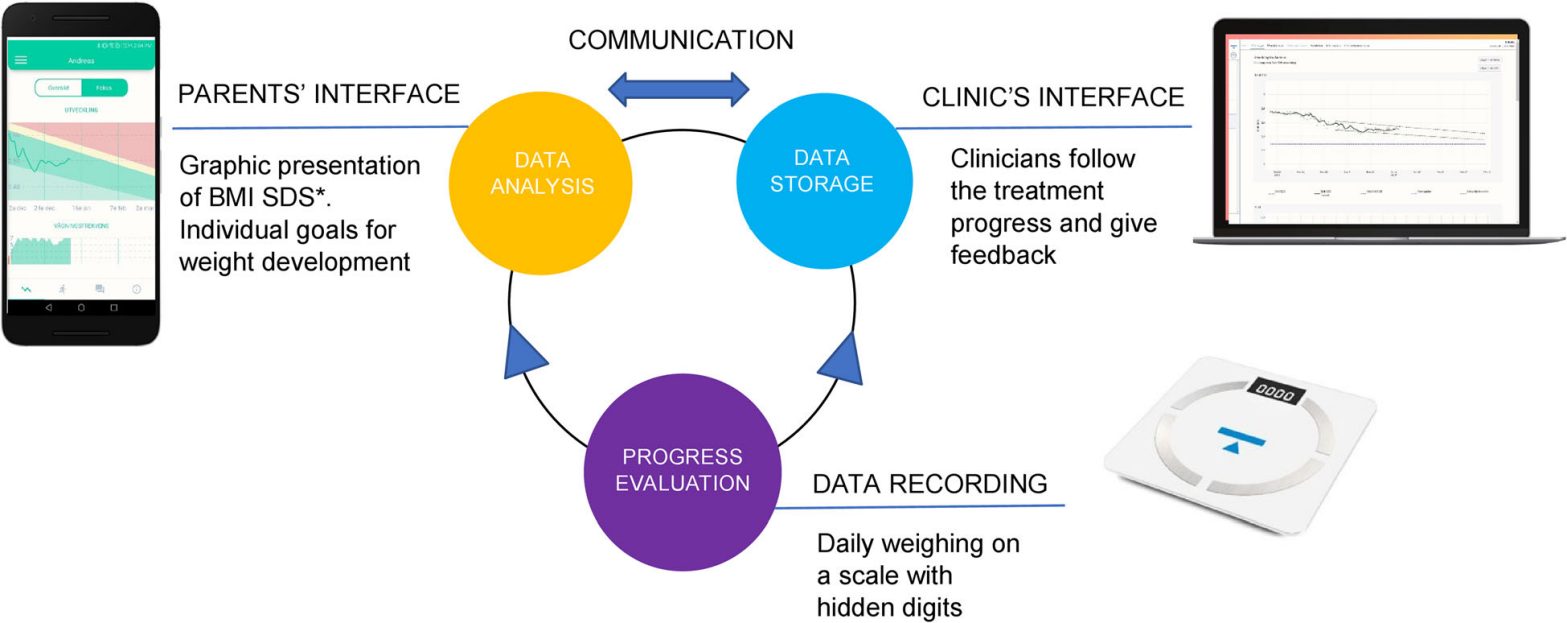
Illustration of the mHealth support system. Layout by Love Marcus. *BMI SDS = Body mass index standard deviation score

The intervention group used an mHealth support system, Provement, developed by Health Support Sweden AB (Stockholm Sweden) (Fig. 1). Parents accessed the support system via a mobile app and clinicians used a website—a clinic’s interface. The mobile app was compatible on Android but not on iOS, therefore, parents using iPhone were provided an Android during the intervention. The app was a prototype that was not commercially available. The mHealth approach comprised daily weighing at home on scales with no displays to indicate weight. Data were transferred via Bluetooth to the mobile app and via a digital cloud server to the clinic’s interface. An individual weight loss target curve was displayed in the mobile app and on the clinic’s interface. The curve included a maximum and minimum value of BMI SDS and the slope of the curve was based on the degree of obesity and estimated growth over the following 3 months. Each curve was manually created by the researchers and the maximum and minimum values of change in BMI SDS generally ranged from − 0.15 to − 0.35 units over 3 months. At 3 months follow-up a new curve was created. On the clinic’s interface clinicians viewed the same data as parents did in the mobile app. Text messages were received and sent from the clinic’s interface for the clinicians, and from the mobile app for the parents.

As an addition to Provement, participants used a commercially available app, Lifee Spirits (Lifee AB, Norrköping, Sweden) to increase motivation for physical activity. A wrist-worn activity monitor was connected to a gamified app via Bluetooth and physical activity generated rewards, in terms of gems and spirits, that were displayed in the app.

At baseline, the parents in the intervention group downloaded the mobile apps and were given scales and activity monitors. Instructions included daily monitoring of the child’s weight and that the activity monitor should be used on the non-dominant wrist daily while awake, except when showering or bathing. Parents were encouraged to follow both apps to receive results on BMI SDS and PA and to write messages whenever they felt a need for support. The clinicians were instructed to check the participants’ weight charts on the clinic’s interface at least weekly and give feedback via text messages.

#### Standard care

In addition to the mHealth approach, the intervention group received standard care, while the control group received standard care alone. Standard care followed the procedure for obesity treatment at each pediatric clinic. All participants met with a clinician (pediatric nurse/dietician/physiotherapist) at the clinic at least every third month. The focus was on lifestyle modification with the aim of improving dietary habits and increasing physical activity to reduce the degree of obesity [22].

### Measures and outcomes

The outcomes of this trial were:

1. Recruitment process i.e. number of eligible individuals, number of randomized participants and reasons for not participating
2. Attrition rates and reasons for attrition
3. mHealth usage i.e. weight frequency at home and messages sent from parents to staff
4. Attendance at appointments in the intervention and the control group
5. Parents’ experience of the mHealth support system at 6 months
6. Parents’ experience of treatment at 6 months, intervention versus control
7. Clinicians’ required working time for the intervention and the control group
8. Clinicians’ experience about the clinic’s interface for the mHealth intervention
9. Changes in BMI SDS [21] in the intervention and the control group after three and 6 months

At baseline, self-reported data on family history of obesity were collected from parents via web-based questionnaires. All parents answered web-based questionnaires on the treatment experience after three and 6 months, with additional questions about the support system for the intervention group and the clinicians. The questionnaires were specifically compiled for this study and contained mostly closed-ended, but a few open-ended questions regarding advantages and disadvantages with the mHealth approach. The questions analyzed in this study are presented in the supplementary material (see Additional files 1 and 2).

The number of measured weights and sent messages among the intervention group was automatically registered on the clinic’s interface. Data about the recruitment process, attrition, attendance, and required working time were documented by the clinicians.

At baseline and follow-up, weight was measured at the pediatric clinics to the nearest 0.1 kg with participants in light clothing (Vetek T1 1200, Sweden; Seca 707, Germany) and height to the nearest 0.1 cm without shoes (Ulmer, Germany; Hyssna, Sweden; Seca, Germany). The scales for self-monitoring of weight, the mobile app, the clinic’s interface and data storage were provided by Health Support Sweden AB (Stockholm, Sweden).

### Participants lost to follow-up and missing data

Participants who attended all follow-up appointments were defined as completers. Participants with no data at 6 months were lost to follow-up, and missing data at 3 months were described as having missing values.

### Statistical analysis

Descriptive data are presented with mean and standard deviation (SD) or with median and interquartile range (IQR) for normal and non-normal distributed variables, respectively. Categorical variables are presented with frequencies and/or percentages. Group differences were calculated using the Student’s t-test, the Chi^2^ test, the Fisher’s exact test, or the Mann Whitney U-test where appropriate. The recruitment process, attrition rates and reasons for attrition are presented narratively and as frequencies. Changes in BMI SDS from baseline to three, and 6 months were evaluated using repeated measures analysis of variance (ANOVA). All participants were included in the analysis and BMI SDS was imputed with the last observation carried forward (LOCF) for participants lost to follow-up, or with missing data. Time specific difference was assessed with Student’s t-test. A *p*-value of < 0.05 was considered statistically significant. IBM SPSS versions 25 and 26 (IBM SPSS Armonk, NY, USA) were used.

## Results

**Fig. 2:**
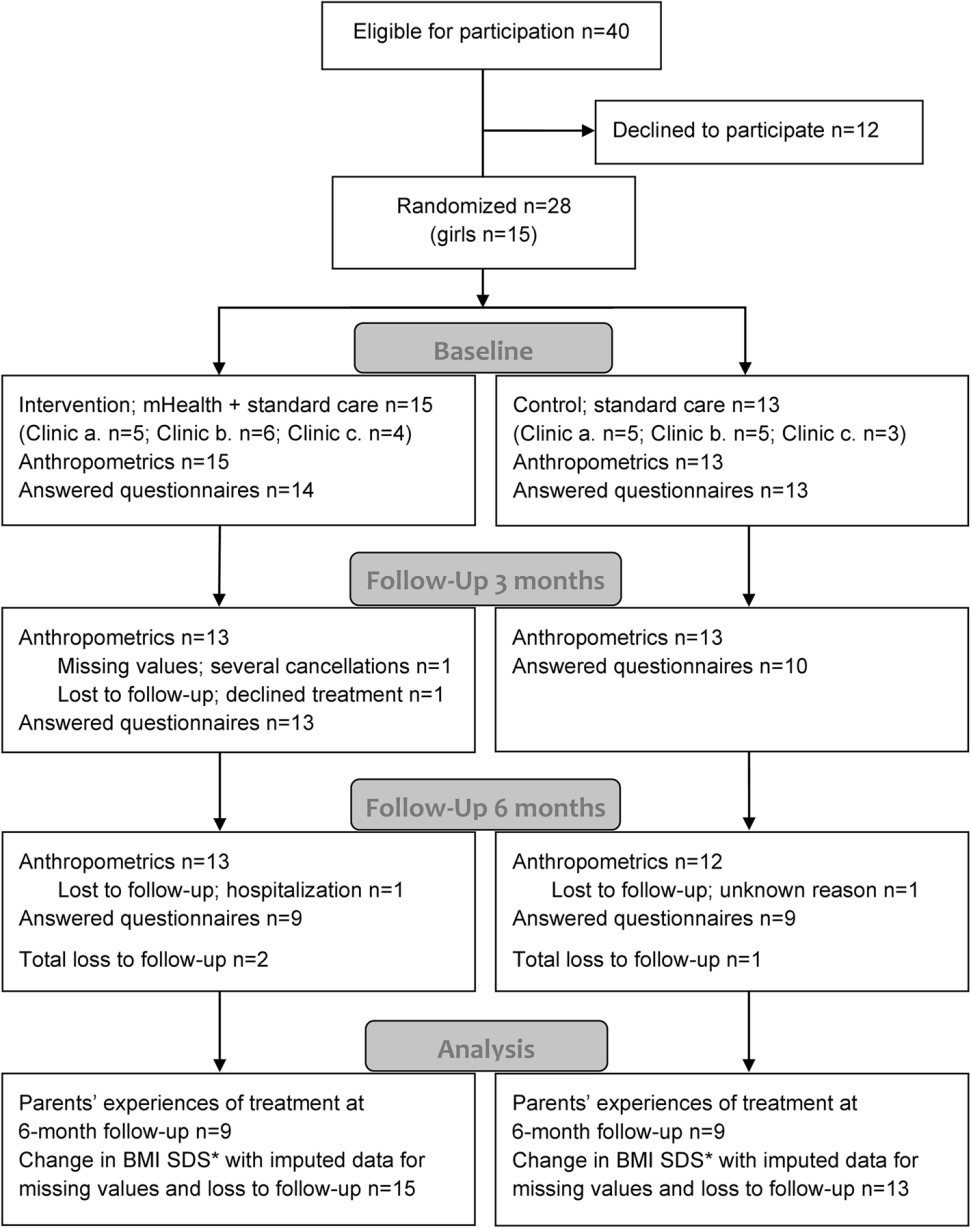
Participant flow chart for allocation and received treatment. Completers and non-completers for baseline and each follow-up are presented together with measured anthropometrics (height and weight) and parents’ response frequency to the questionnaires about their expectations and treatment experience. *BMI SDS = Body mass index standard deviation score

During the recruiting period, 40 children were eligible and 28 participants (15 girls) agreed to participate in the trial and were randomized. Reasons for not participating included skepticism about daily weighing and lack of motivation. In the intervention group, two children were lost to follow-up. One child stop participating because of technical difficulties with the mobile app and one child was hospitalized for reasons unrelated to obesity. One participant in the control group was lost to follow-up for unknown reasons (Fig. 2). The baseline characteristics between the two groups were similar (Table 1).

**Table 1:** Child and parent characteristics at baseline

Variable	Intervention *n* = 15	Control *n* = 13	*P*^1^
Girls n (%)	9 (60)	6 (46)	0.464
Age, mean [sd] (min–max)	8.4 [1.9] (5.2–11.2)	9.8 [2.2] (5.1–12.8)	0.083
Height in cm, mean [sd] (min–max)	138.0 [14.3] (108.8–156.4)	148.2 [16.2] (118.3–170.0)	0.087
Weight in kg, mean [sd] (min–max)	50.0 [13.4] (25.0–70.0)	61.5 [22.6] (29.0–96.1)	0.124
BMI, mean [sd] (min–max)	25.7 [3.3] (20.3–31.6)	27.0 [4.5] (20.7–33.3)	0.392
BMI SDS, mean [sd] (min–max)	3.0 [0.5] (2.2–4.2)	2.8 [0.3] (2.2–3.1)	0.189
Degree of obesity^2^			0.431
Severe Obesity n (%)	8 (53)	5 (38)	
Obesity n (%)	6 (40)	7 (54)	
Overweight n (%)^3^	1 (7)	1 (8)	
Non-Nordic origin n (%)^4^	8 (53)	5 (39)	0.431
**Self-reported parental data**	**Intervention** ***N*** **= 15**	**Control** ***N*** **= 12**	
One parent has/has had obesity n (%)^5^	10 (67)	10 (83)	0.408
Two parents have/have had obesity n (%)	3 (20)	4 (33)	0.662
One parent has had obesity surgery n (%)^6^	1 (7)	1 (8)	1.00

^1^*p-*values derive from Student’s t-test for continuous variables and from chi-squared test or Fisher’s exact test for other variables. An alfa of < 0.05 is considered statistically significant

^2^For group differences, participants were categorized as having either severe obesity or obesity/overweight

^3^All children had obesity at inclusion but two individuals were classified as overweight at baseline

^4^At least one parent was born in a non-Nordic country

^5^At least one parent has or has had obesity

^6^At least one parent has had obesity surgery

### mHealth usage

**Fig. 3:**
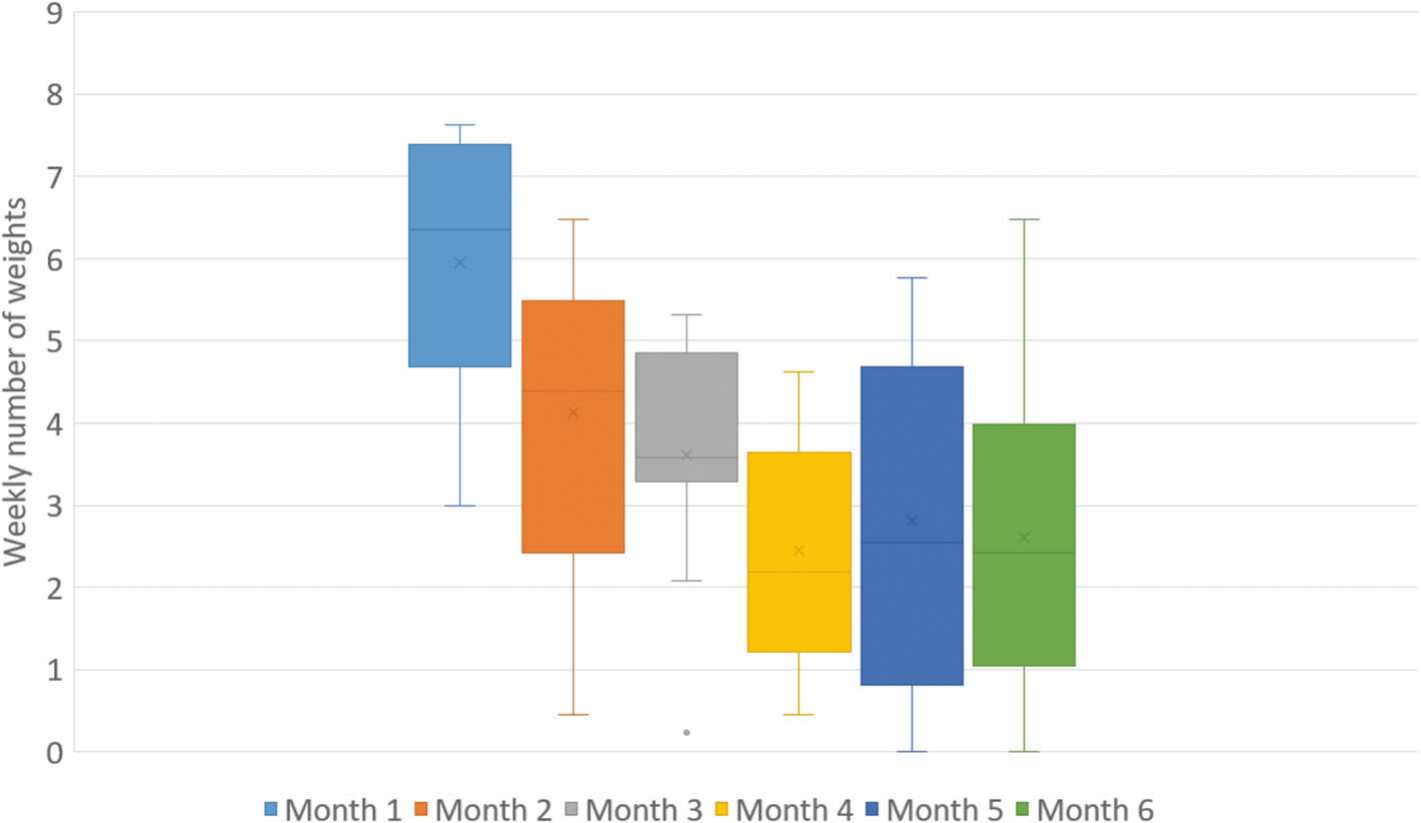
The weekly number of weights per month, for study completers using the mHealth support system (*N* = 12). The box illustrates the mean value(x) and the median (line) with the first and third quartile. The whiskers show the minimum and maximum values

Among the intervention group, 5/15 parents had to borrow an Android cell phone. Two children were lost to follow-up and one child lacked motivation and stopped using the scale after 6 weeks but attended the follow-up appointment at 6 months. Hence, weight was measured at home regularly throughout the entire study period by 12/15 children (completers). Weight frequency, among completers, was highest during the first month with a mean (SD) of 5.9 (1.6) measurements per week and a stabilization with approximately 2.4 (1.4) measurements per week from month four and onwards (Fig. 3). Among completers messages were sent with a median (IQR) frequency of 4 (6) messages and sent messages ranged between 0 and 13.

### Compliance with treatment

More children in standard care alone (85%) cancelled at least one appointment compared to the intervention group (40%) (*p* = 0.024). Non-attendance of at least one planned appointment tended to be more frequent in the control group (4/13 children) than the intervention group (1/15 children), *p* = 0.153.

### Parents’ acceptability of treatment

In the intervention group, 89% of the parents reported that the support system helped them reach the treatment goal and that the system made it easier getting in touch with the clinicians quickly. The feedback from clinicians was considered satisfactory, and the weight loss target curves were considered helpful in 67% of the families. The attitude towards daily weighing was positive or neutral in 89% of the parents. However, 11% reported difficulties in remembering or motivating their child to step on the scale. One third of the parents would have preferred that the scales for daily weighing had displayed weight and two thirds disagreed or were neutral. The most frequently reported issue was technical difficulties with the prototype of the support system. Other challenges were resistance from the child towards daily weighing and loss of interest when weight gain occurred.

All parents reported that they followed their child’s physical activity through the gamified application and 78% reported that their child wore the activity monitor on most days. Of the parents, 56% stated that the monitor was fun to use for their child, although 67% did not have the impression that it resulted in increased PA.

The parents in the intervention group were satisfied with the treatment results (78%) to a higher extent than the control group (11%) (*p* = 0.015). The parents’ expectations of the obesity treatment were met in 78% of the intervention group and in 22% of the control group (*p* = 0.057).

### Clinicians’ acceptability of treatment

At the three pediatric clinics, four clinicians used the interface of the support system. All clinicians reported that they logged in to the interface once a week. The message function was mainly used for feedback on weight development and reminders about weighing. Challenges included technical difficulties and that the curves for weight development were difficult to understand. However, 2/4 clinicians reported that they did not experience any challenges with the system. All clinicians stated that the most prominent benefit of the intervention was its contribution to a clear treatment goal, 75% of the clinicians thought it enhanced the possibility of following the patient’s weight development, and 50% stated that the support system facilitated communication with the parents.

Clinicians spent more time on children in the intervention than the control group (*p* = 0.002). The number of appointments, excluding baseline, and time spent by clinicians on treatment, is presented in Table 2.

**Table 2:** Number of follow-up appointments, phone calls, text messages and time spent by clinicians on treatment

Variable	Intervention *n* = 15	Control *n* = 13	*P*^1^
Number of appointments, median [IQR] (min–max)^2^	2.0 [1.0] (0–5)^3^	2.0 [0.0] (1–3)	0.274
Number of phone calls, median [IQR] (min–max)	1.0 [3.0] (0–9)	2.0 [2.0] (0–5)	0.683
Number of text messages sent from staff, median [IQR] (min–max)	13.0 [10.0] (4–23)	N/A	N/A
Minutes for appointments and documentation, median [IQR] (min–max)	140.0 [70.0] (0–300)	120.0 [40.0] (60–180)	0.156
Minutes on phone calls and documentation, median [IQR] (min–max)	10.0 [40.0] (0–140)	10.0 [12.5] (0–60)	0.928
Minutes on text messages sent by staff, median [IQR] (min–max)	70.0 [59.0] (20–115)	N/A	N/A
Minutes on all contacts and documentation, median [IQR] (min–max)	215.0 [97.0] (45–526)	120.0 [67.5] (75–240)	0.002^a^

^1^*p* values are based on a Mann Whitney U-test

^2^Follow-up appointments, not including baseline

^3^One child was lost to follow-up after 2 months – therefore no follow-up appointments

^a^An alfa of < 0.05 is considered statistically significant

### Changes in BMI SDS

**Fig. 4:**
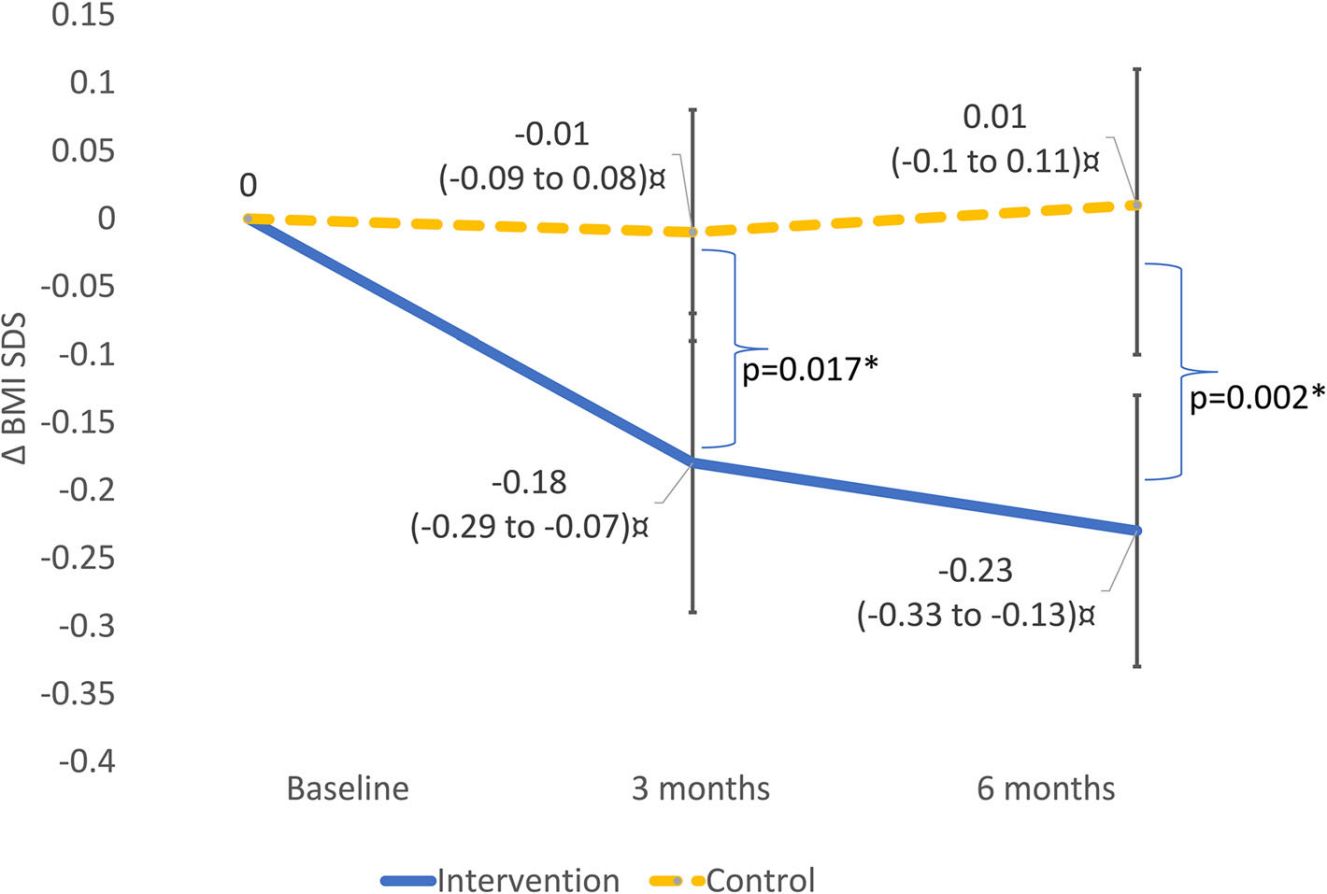
Changes in BMI SDS from baseline to follow-up at three and 6 months for all study subjects (intervention *n* = 15, control *n* = 13).^**¤**^ Mean change in BMI SDS, with 95% confidence interval. *A *p*-value of < 0.05 was statistically significant. *P*-values are based on Student’s t-test

The repeated measures ANOVA showed significantly better results in the intervention group compared to the control group (*p* = 0.004). At the three-month and six-month follow-up, the mean differences of BMI SDS between the groups were 0.17 and 0.24 units, respectively (Fig. 4). The number of treatment responders, i.e. participants with no increase in BMI SDS, was greater among the intervention group (14/15) than the control group (6/13). The non-responder in the intervention group stopped using the support system after 6 weeks.

## Discussion

This is the first randomized controlled feasibility trial to investigate an mHealth support system in pediatric obesity treatment combining collection of objectively measured weight data, treatment goals and results of relative weight change displayed in a mobile app, and communication via text messages between parents and clinicians. Both parents and clinicians had a positive experience and found the support system accessible. Additionally, parents and clinicians reported that the distinct individual treatment goal was beneficial for the families involved.

In the recruitment process, 30% of the families declined to participate, partly because of their skepticism towards daily weighing. An apparent concern among parents and clinicians is whether childhood obesity treatment increases the risk of eating disorders (ED). However, no such associations were found in a systematic review and meta-analysis by Jebeile et al., which included 2589 children. Instead, they found a reduction in ED risk and symptoms when the children attended structured treatment [23].

### mHealth usage and compliance

Most children in the intervention group measured their weight regularly, although, weight frequency decreased during the first 3 months. Accordingly, Wing et al. [24] have shown that self-monitoring of weight in adults decrease over time. However, participants measuring weight daily managed to maintain weight loss better than those with a lower weight frequency [24]. Receiving daily feedback via text messages seem to increase the frequency of self-monitoring in adults, even though the feedback did not affect weight change [25]. In a study by Bala et al. [26] parents to children with obesity received messages about self-monitoring and behavioral change twice a week. Parents were satisfied with the content of the received messages, however, the response rate was low with 40% of the parents not answering more than half of the received messages [26]. The same trend was seen in the present study, in which parents received far more messages than they wrote. It might be unrealistic to expect that all parents are interested in interactive communication through text message as a part of the obesity intervention.

The parents in the intervention group were more satisfied with the treatment and the results than the control group. Low satisfaction and perceived quality of care are indicators of attrition in pediatric obesity treatment [27] and accessible approaches, for example through mHealth, may be potential solutions [6, 28]. The drop-out rate in the present study was low, although participants, primarily in the control group, frequently cancelled appointments. Compared to standard care alone, Armstrong et al. found that children who received additional text messages had better adherence to appointments [29]. Similarly, the intervention group had a smaller number of cancellations, indicating that the support system reduced barriers to face-to-face meetings.

### Treatment acceptability

There is a risk that both parents and children may perceive daily weighing as being a stressful aspect of the treatment [30]. Nevertheless, most parents who used the support system reported that measuring weight at home went well. To the best of our knowledge, no studies have been published on the psychological effects and self-weighing in younger children. In adults and young adults, several robust studies have found no associations between self-monitoring of weight and negative psychological effects. On the contrary, self-weighing has been associated with a BMI reduction [31–34] and would appear to be an important success factor in obesity treatment [35].

In the present study, most parents had the impression that the activity monitor and gamified app was fun to use for their child, but that it did not result in increased PA. This is in accordance with a study by Direito et al. in which children used a gamified app for 8 weeks [36]. One of the key issues with gamification is that continuous updates of the games would appear to be necessary in order to keep the participants motivated [37].

Not surprisingly, the total required working time of the clinicians was higher for the intervention group. Since this mHealth support system has not been previously evaluated, it was chosen to serve as an addition to standard care for ethical reasons. However, the healthcare staff spent a minimal amount of time (5 min) on each digital consultation, indicating that the system could be time effective when used more frequently at the clinic.

### Changes in BMI SDS and potential harms of treatment

The intervention group reduced its BMI SDS drastically, while children in standard care increased to some extent after 6 months of treatment. Changes in BMI SDS by at least − 0.25 units after 12 months of treatment have been shown to result in improved cardiometabolic parameters and has therefore been suggested as a clinically relevant treatment outcome [38]. Nonetheless, most approaches in behavioral childhood obesity treatment fail to meet this criterion [39, 40]. Accordingly, the results of the current study show no treatment effects on children who received standard care. However, the results for children in the intervention group were close to clinical relevance, even after only 6 months.

Childhood obesity is a disease associated with severe long-term consequences [41, 42] . Reduction of the degree of obesity is stressful for most people and, like all medical treatments, the negative effects of the treatment need to be balanced against the benefits. Daily monitoring of weight and text messages between parents and clinicians may result in early detection of the potential harm of treatment. However, in this study there were no specific outcome measures regarding psychological effects, stress and eating disorders, therefore, the risk should be further evaluated in a study with a larger number of participants.

### Limitations and strengths

One limitation of this study is that only parents and clinicians, and not the child, were asked to answer the questionnaires about their treatment experience. Further, due to the novelty of this treatment approach, the questionnaires were not validated. This trial only included children that did not receive obesity treatment during the last 6 months. To improve external validity future studies should also include children with an ongoing treatment.

The main strength of this study is that all weight data were measured objectively. Another strength is that although a new technological intervention was studied, there were many completers—which is a rare phenomenon among children in obesity treatment.

### Future prospects

Future research of the evaluated support system should make the following adoptions to improve quality: a) the weight loss target curves should be automatically generated, b) the app should be made available for several operating systems and, c) a validated activity monitor should be directly connected to Provement. Further, future research should evaluate the child’s perspective, potential harm, and the treatment effects over a longer follow-up time in a larger controlled study.

## Conclusions

The evaluated mHealth approach was acceptable and positively received by both parents and clinicians. This intervention provided an innovative treatment approach which, in addition to standard care, generated better treatment results than standard care alone. We believe that the support system could help identify non-responders and non-compliers and thereby direct resources, i.e. more appointments, where they are needed most. In parallel, those patients responding to treatment could have less appointments, leading to less absence from school and work for the families involved.

## Supplementary information

**Additional file 1.** Parts of a web-based questionnaire addressed to parents after 3 and 6 months of treatment. This additional file includes the questions used for presenting the results in this manuscript. The questions are originally written in Swedish.

**Additional file 2.** Parts of a web-based questionnaire addressed to clinicians after 3 and 6 months of treatment. This additional file includes the questions used for presenting the results in this manuscript. The questions are originally written in Swedish.

## Data Availability

Due to the small number of participants at each clinic, data can indirectly be traced back to the study participants. Therefore, the data sets generated and analyzed in the current study are not publicly available. Request of data access should be addressed to the corresponding author and will be handled on a case by case basis.

## Abbreviations

mHealth: Mobile health
app: Application
PA: Physical activity
BMI: Body mass index
SDS: Standard deviation score
SD: Standard deviation
IQR: Interquartile range
ANOVA: Analysis of variance
LOCF: Last observation carried forward
CI: Confidence interval
