# Supplementary material for: A novel interactive mobile health support system for pediatric obesity treatment: a randomized controlled feasibility trial
Source: BMC Pediatr. 2020 Sep 23;20:447. doi: 10.1186/s12887-020-02338-9 (PMC7513491; doi:10.1186/s12887-020-02338-9)
Supplement: Supplementary file 1 — Additional file 1. Parts of a web-based questionnaire addressed to parents after 3 and 6 months of treatment. This additional file includes the questions used for presenting the results in this manuscript. The questions are originally written in Swedish. [file 12887_2020_2338_MOESM1_ESM.pdf]

# Parts of a web-based questionnaire addressed to parents after 3 and 6 months of treatment

## The presented questions, translated from Swedish to English, are data used in the manuscript

## Parents in the intervention group and the control group were asked the following questions:

1. **Did the received childhood obesity treatment meet your expectations?**

a) Yes, completely
b) Yes, almost completely
} Yes completely/almost completely (categorized for analysis)
c) Partly
d) Marginally
e) No, not at all
} Partly/Marginally/Not at all (categorized for analysis)

2. **Are you satisfied with your child's weight development?**

a) Yes, completely
b) Yes, almost completely
} Yes completely/almost completely (categorized for analysis)
c) Partly
d) Marginally
e) No, not at all
} Partly/Marginally/Not at all (categorized for analysis)

## Parents in the intervention group were asked the following questions:

3. **Did the mHealth application help you reaching the treatment goal?**

a) Yes, completely
b) Yes, almost completely
c) Partly
d) Marginally
e) No, not at all

4. **Did the mHealth application make it easier to get in touch with the clinicians quickly?**

a) Yes, completely
b) Yes, almost completely
c) Partly
d) Marginally
e) No, not at all

5. **What was your experience of measuring weight daily?**

a) Very good
b) Quite good
c) Neither good nor bad
d) Quite difficult
e) Very difficult

6. **Were the text messages sent from the clinicians useful?**
a) Yes, completely
b) Yes, almost completely
c) Partly
d) Marginally
e) No, not at all

7. **Were the weight loss target curves helpful to you?**
a) Yes, very helpful
b) Yes, quite helpful
c) Partly helpful
d) Marginally helpful
e) No, not at all helpful
f) I did not understand the weight loss target curves

8. **Would it be better if the scale for daily weighing had displayed the weight?**
a) Yes, definitely
b) Perhaps
c) No opinion
d) Not likely
e) Absolutely not

9. **Which were the greatest advantages with the mHealth app?**
*Optional comment*

10. **Which were the greatest disadvantages with the mHealth app?**
*Optional comment*

11. **Did your child remember to wear the activity monitor every day?**
a) Yes, always
b) Almost always
c) Partly
d) Marginally
e) No, not at all

12. **Did your child find the activity monitor fun to use?**
a) Yes, very fun
b) Yes, quite fun
c) Either or
d) Not so much fun
e) Not fun at all

13. **Did you look at the app for physical activity together with your child?**
a) Yes, always
b) Almost always
c) Partly
d) Marginally
e) No, not at all

**14. Do you have the impression that your child became more physically active when using the activity monitor?**

a) Yes, completely
b) Yes, almost completely
c) Partly
d) Marginally
e) No, not at all
