# Supplementary material for: A novel interactive mobile health support system for pediatric obesity treatment: a randomized controlled feasibility trial
Source: BMC Pediatr. 2020 Sep 23;20:447. doi: 10.1186/s12887-020-02338-9 (PMC7513491; doi:10.1186/s12887-020-02338-9)
Supplement: Supplementary file 2 — Additional file 2. Parts of a web-based questionnaire addressed to clinicians after 3 and 6 months of treatment. This additional file includes the questions used for presenting the results in this manuscript. The questions are originally written in Swedish. [file 12887_2020_2338_MOESM2_ESM.pdf]

Parts of a web-based questionnaire addressed to clinicians after 3 and 6 months of treatment

The presented questions, translated from Swedish to English, are data used in the manuscript

1. How often did you log in to the clinic's interface?

a) Every day
b) Several times a week
c) One time a week
d) Less often than one time a week

2. Which topics were most commonly addressed in the messages you sent to the parents? Multiple response options are possible

a) Reminders about weighing
b) Reminders about using the activity monitor
c) Feedback regarding relative weight change
d) Feedback regarding physical activity
e) Dietary advice
f) Physical activity advice
g) Scheduling of appointments
h) Other topics

3. Did you experience any difficulties with the clinic's interface?

a) Yes
b) No

4. If your response to the previous question was 'yes', which difficulties did you experience? Multiple response options possible

a) Technical difficulties
b) Vague message function
c) Time consuming
d) The weight loss target curves were difficult to understand

5. In your opinion, which were the major advantages with the mHealth support system/the clinic's interface? Multiple response options possible

a) Facilitates the communication with the parents
b) Provides a clear treatment goal
c) Makes it easy to track the patient's weight development
d) Time saving e.g. through fewer appointments
